# Kommunikation des Evangeliums und Populäre Religion. Annäherung an ein Spannungsverhältnis am Beispiel des YouTube-Kanals „Jana glaubt“

**DOI:** 10.1007/s41682-021-00067-z

**Published:** 2021-06-25

**Authors:** Jonathan Kühn, Henrik Simojoki

**Affiliations:** Humboldt-Universität zu Berlin, Berlin, Deutschland

**Keywords:** Populäre Religion, Kommunikation des Evangeliums, Mediatisierung von Religion, Evangelische Kirche in Deutschland, YouTube Kanal „Jana glaubt“, Popular Religion, Communication of the Gospel, Mediatization, Protestant Church in Germany, YouTube channel “Jana believes”

## Abstract

Das Verhältnis von Religion und populärer Kultur hat in der deutschsprachigen Religionssoziologie breite Aufmerksamkeit gefunden, ist aber bislang nur selten für die Untersuchung kirchlich verfasster Religionspraxis fruchtbar gemacht worden. Mit dem von der EKD mitgetragenen Online-Kanal „Jana glaubt“ erschließt der vorliegende Beitrag ein profiliertes und auch kontrovers diskutiertes Pionierprojekt im Schnittfeld von Populärer Religion und kirchlicher Glaubenskommunikation.

Die Argumentationsführung erfolgt in sechs Schritten. Zunächst werden die für den Beitrag leitenden Theorieperspektiven – das von Hubert Knoblauch entwickelte Konzept Populärer Religion und der praktisch-theologische Leitbegriff der Kommunikation des Evangeliums – zusammengeführt und mit dem in der interdisziplinären Religionsforschung zugkräftigen Mediatisierungsansatz verbunden. Anschließend werden populärkulturelle Eigenlogiken von YouTube-Kommunikation expliziert, damit vor diesem Hintergrund die Adaption des Influencing-Genres im Kanal „Jana glaubt“ in ihrer Spezifizität erhellt werden kann. Weiter konkretisiert wird der Zugriff auf das Zusammenspiel von Populärer Religion, Kommunikation des Evangeliums und Mediatisierung in zwei exemplarischen Materialanalysen, die sich forschungsmethodisch an Jürgen Raabs Analysekonzept einer Visuellen Wissenssoziologie orientieren.

Der Beitrag mündet in einer Zusammenfassung, die beide Seiten des erschlossenen Spannungsverhältnisses bündelt: Auf der einen Seite entsprechen die Gestaltungselemente in den Kanalvideos etablierten Standards personalisierter YouTube-Kommunikation. Zudem werden in den Video-Analysen für Populäre Religion grundlegende Dynamiken der Entgrenzung manifest: Kommunikation des Evangeliums wird öffentlich, indem das Private geteilt wird – in der Erwartung, dass gerade die subjektive Darstellung des Privaten den christlichen Glauben kommunikativ anschlussfähig macht. Auf der anderen Seite wirkt sich die kirchliche Einbettung transformativ auf die populärreligiösen Darstellungspraktiken aus. Dabei kommt es besonders bei Fragen der Authentizität und der Autorität zu teils konflikthaften Aushandlungsprozessen.

## Abstract (en)

The relationship between religion and popular culture has attracted considerable attention in the field of the sociology of religion. However, church-based religious communication has rarely been explored in this perspective. This in mind, the paper explores the YouTube channel “Jana believes”, a pioneering and also controversial project sponsored by the Evangelical Church in Germany.

The argumentation proceeds in five steps. First, Hubert Knoblauch’s concept of “popular religion” is interlinked with the practical-theological perspective of “communicating the Gospel” as well as with more recent approaches to the mediatization of religion. Then characteristic elements and video genres of YouTube communication as part of popular culture are explicated. Against this background, the distinct profile of the YouTube channel “Jana believes” is outlined, with specific regard to the vlog genre. Subsequently, the interplay between popular religion, communicating the Gospel and mediatization of religion is explored in a more in depth-analysis of two exemplary video sequences.

Finally, the summary brings together both sides of the interplay in question: On the one hand, the channel videos are designed according to established standards of personalized YouTube communication. In line with the concept of popular religion, the boundaries between private and public become fluid. Public communication of the Gospel is constituted by sharing what is private in life and faith. On the other hand, in the case of this channel the subjective enactment of faith is also shaped and transformed by the involvement of the church. Particularly regarding questions of authenticity and authority, the tension between popular religion and communicating the Gospel at times leads to potentially conflictual negotiation processes.

## „Gottes Influencerin“ – Spannungsmomente einer kirchlichen Digitalinitiative

Am 13. April 2018 war es endlich so weit: Nach aufwändigen Vorbereitungen wurde die erste Folge des YouTube-Kanals „Jana glaubt“ hochgeladen. Damit machte sich der kirchliche Protestantismus in Deutschland erstmals konsequent und unter Einsatz erheblicher Mittel ein digitales Medienformat zu eigen, das sich einen festen Platz im Medienalltag vieler deutscher Jugendlicher und junger Erwachsener erobert hatte: Im Jahr der Einführung von „Jana glaubt“ war der Kanal „Bibis Beauty Palace“ der Influencerin Bianca Claußen mit gleich drei Beiträgen bei den erfolgreichsten YouTube-Videos vertreten; das populärste Video kam auf 6,6 Mio. Aufrufe.^1^ So war die Entscheidung, sich als Kirche auf dieses Terrain zu wagen, durchaus folgerichtig, zumal mit dem Projekt 14- bis 29-Jährige erreicht werden sollten. Bis zum Einstellen des Kanals inmitten der ersten Welle der COVID-19-Pandemie wurden 185 Videos produziert. Hinzu kam ein umfangreiches Begleitangebot in sozialen Netzwerken wie Instagram und Facebook.

Im Zentrum des Kanals stand Jana Highholder, eine zum Programmstart 19-jährige Medizinstudentin und Poetry-Slammerin aus Münster. Trotz aller gattungstypischen Personalisierung handelte es sich aber um keinen Privatkanal. „Jana glaubt“ wurde im Auftrag der Evangelischen Kirche in Deutschland (EKD) gemeinsam vom Gemeinschaftswerk der Evangelischen Publizistik (GEP) und der Arbeitsgemeinschaft der Evangelischen Jugend in Deutschland (aej) verantwortet. Produziert wurden die Formate allerdings von einem externen Dienstleister. Besucht man die Homepage der Agentur, findet man folgende Selbstbeschreibung:

MEDIAKRAFT NETWORKS ist die Modern Media Company des social-first Zeitalters. Wir sind Spezialisten für nachhaltiges Content- und Influencer-Marketing und unterstützen Marken sowie Creator dabei, junge Zielgruppen mit relevanten und unterhaltsamen Inhalten zu erreichen. Beratungsstark, kreativ und umsetzungserfahren gestalten wir seit 2011 mit unseren Online-Video-Formaten die Zukunft des Brand Storytellings aktiv mit.^2^

Angesichts der Marketingsprache überrascht es nicht, dass dieses Format in der medialen Öffentlichkeit und im pluriformen Feld des kirchlichen Protestantismus auf ein geteiltes Echo stieß. Die Frage, an der sich die Geister schieden, lautete: Ist das die richtige Antwort auf das digitale Zeitalter? Allerdings richtete sich diese Frage weniger auf die Orientierung der EKD am marktförmigen Influencing-Genre als vielmehr auf die religiösen Position der Protagonistin: Jana Higholder hat einen evangelikal-freikirchlichen Hintergrund und hielt mit ihren Überzeugungen auch dann nicht hinterm Berg, wenn sie, etwa im Blick auf das Familien- und Frauenbild, mit der Grundrichtung von EKD-Positionen kollidierten.^3^ Zudem ist sie nicht theologisch ausgebildet, was ebenfalls nach Ansicht mancher Kritikerinnen und Kritiker in deutlicher Spannung zum evangelischen Grundverständnis der an ein kirchliches Amt gebundenen öffentlichen Verkündigung steht (vgl. Leitlein et al. 2019). Interessanterweise kommt die Begründungsfigur des Allgemeinen Priestertums, auf der das evangelische Amtsverständnis ja theologisch aufruht, in der Diskussion kaum zum Tragen.

Ihre persönliche Website enthielt auch während des Projektzeitlaufs keine Bezüge zur EKD. Im Mai 2020 stellte sie sich und ihre Mission dort folgendermaßen vor:

Ich bin Jana. Gottes Influencerin. Im Oktober 2014 habe ich begonnen, als Poetry Slammerin auf den deutschsprachigen Bühnen zu stehen und mit wortgewaltigen Texten Menschen und Herzen zu begeistern und bewegen. Mittlerweile bin ich Autorin von drei Büchern und Gesicht des Youtube Kanals „Jana glaubt“; einem Projekt, das Glaube zugänglich machen und erklären will. Ich möchte den Menschen dort begegnen, wo sie sind – und in meiner Generation sind das die sozialen Medien. Dort nehme ich euch mit in mein Leben zwischen Medizinstudium, Bühne, Alltag und zeige, dass Glauben nicht von gestern, sondern für Heute und Morgen ist. Wenn wir auf Social Media alles finden, warum dann nicht die beste Botschaft der Welt? Ich nutze meine Reichweite, um von Gott zu erzählen. Er ist nicht gebunden an alte Bücher und dicke Mauern, er lässt sich finden – auch auf Instagram, auch auf Youtube. Dort bin ich unterwegs als „Gottes Influencerin“. Meine Vision ist eine Jugend, die Gott kennt. Eine Jugend mit Gott.^4^

In diesem Zitat bricht ein Spannungsverhältnis im Schnittfeld von Theologie und Religionssoziologie auf, das im vorliegenden Beitrag ausgelotet werden soll. Der eine Pol dieses Spannungsfeldes verdichtet sich in der bereits in den 1960er-Jahren eingeführten Formel der „Kommunikation des Evangeliums“, die sich in Kirche und Theologie als „Modalbestimmung christlicher Religionskultur“ (Engemann 2014, S. 17) etabliert hat und gleichzeitig aufgrund analytischer Blickwinkelverengungen bis heute umstritten geblieben ist. Der andere Pol wird durch das von Hubert Knoblauch (2008, 2009) maßgeblich konturierte Konzept der „Populären Religion“ markiert, das im Fokus dieses Sonderheftes steht. Auf den ersten Blick scheint die beiden Perspektiven wenig zu verbinden: Die eine entstammt dem praktisch-theologischen Diskurs, setzt eine christliche Wirklichkeitssicht voraus und wird zumeist auf Formen kirchlicher Glaubenskommunikation bezogen; bei der anderen handelt es sich um einen soziologischen Diagnosebegriff, der das religiöse Feld aus der Außenperspektive erschließt und dabei – in Fortschreibung des Individualisierungsparadigmas – vom kirchlichen Christentum entkoppelte Sozialformen des Religiösen in besonderer Weise im Blick hat. Bei näherem Hinsehen zeigen sich jedoch vielfältige Überlappungen, die am Beispiel des YouTube-Kanals „Jana glaubt“ plastisch zutage treten. Um diese Überlappungen schärfer zu erfassen, müssen jedoch beide Perspektiven theoretisch verlinkt werden, mit besonderem Augenmerk auf Mediatisierungsdynamiken im Kontext digitaler Religionskommunikation. Sodann sollen sie auch anhand exemplarischer Materialanalysen empirisch konkretisiert werden. Wie sich dabei zeigen wird, ist der kirchlich initiierte YouTube-Kanal mit und rund um Jana Highholder nicht allein ein kirchliches Pionierprojekt im Kontext der Digitalisierung, sondern zugleich ein Beispiel populärreligiöser Entgrenzungsphänomene.

## Populäre Religion, Kommunikation des Evangeliums und die Mediatisierung des Religiösen. Versuch einer diskursiven Verlinkung

Der YouTube-Kanal „Jana glaubt“ als Plattform christlicher Glaubenskommunikation ist eingebettet in einen gesellschaftlichen, medialen und kulturellen Kontext, den Hubert Knoblauch (2008, 2009) umfassend analysiert und konzeptualisiert hat. Demnach hat sich ab den 1960er-Jahren in vielen westlichen Industriegesellschaften eine grundlegende Transformation des Religiösen vollzogen, die sich als eine weitgehende Spiritualisierung von Religion begreifen lässt. Ob innerhalb oder außerhalb religiöser Organisationen anzutreffen, zeichnen eine solche Spiritualität in soziologischer Hinsicht neben einer in der Tendenz antiinstitutionellen wie auch antidogmatischen Einstellung insbesondere Popularisierung, Ganzheitlichkeit sowie ein entschiedener Subjektivismus mit ausgeprägter Erfahrungsbetontheit aus (Knoblauch 2009, S. 124).

Natürlich sind solche Spiritualisierungsdynamiken frömmigkeitsgeschichtlich nicht neu – in der Geschichte des Christentums sind sie etwa in mystischen und asketischen Strömungen präsent (für den Protestantismus vgl. die klassische Analyse von Troeltsch 1912, S. 848–964). So betont auch Knoblauch die Kontinuität der von ihm rekonstruierten Spiritualisierungsphänomene zu der von Troeltsch als „Mystik“ bezeichneten Sozialform des Religiösen (Knoblauch 2009, S. 125 f.). Im Zuge der funktionalen Differenzierung, der Entwicklung hin zur Wissensgesellschaft und schließlich auch der mit der Digitalisierung verbundenen Individualisierungsschübe habe aber die dogmatische und organisatorische Distanz zum vormals „offiziellen“ Modell von Religion etwa des großkirchlichen Christentums an Breite und mehrheitskultureller Plausibilität gewonnen.

Bis hier bleibt Knoblauch noch weitgehend in den Bahnen der Privatisierungstheorie seines Lehrers Thomas Luckmann. Allerdings trägt er in seiner Diagnose der „Refiguration der Religion“ (Knoblauch 2020) etliche Modifikationen ein, die im Sinne der Leitperspektive dieses Sonderheftes auf neuartige Dynamiken der „Entgrenzung“ verweisen und von Knoblauch unter dem Oberbegriff einer „Populären Religion“ entfaltet werden. Die Populäre Religion kann in ihren Konkretionen UFO-Glauben ebenso einschließen wie Marienerscheinungen, eventisierte Zeremonien im Zusammenhang mit Papstbesuchen, Wünschelrutengehen oder noch relativ junge gesellschaftliche Praxen wie die mit Halloween verbundenen Bräuche. Sie ist „die kulturelle Ausdrucksweise der neuen Spiritualität“ (Knoblauch 2008, S. 6) und konstitutiv davon bestimmt, dass jedes Individuum Transzendenzerfahrungen machen kann. Im Blick auf das Analysebeispiel dieses Beitrages sind folgende Aspekte der Entgrenzung von besonderer Bedeutung:

- Bekanntlich ist die Transformation von Religion in der Moderne bei Luckmann mit einer grundlegenden Ortsverschiebung verbunden: Da Religion keiner institutionellen Vermittlung mehr bedarf, verlagert sie sich aus der Öffentlichkeit in den privaten Raum. Dagegen verweist Knoblauch wiederum darauf, dass individualisierte Religionsformen in der spirituellen Gesellschaft massenkulturelle Sichtbarkeit gewinnen. Die Refiguration von Religion in spätmodernen Gesellschaften führt damit weder zu einer „unsichtbaren“ noch zu einer „öffentlichen Religion“, sondern „hat eine Auflösung der Grenzen zwischen der Privatsphäre und der Religion zur Folge: Das Öffentliche steht nicht mehr im Kontrast zum Privaten, das Private kann vielmehr öffentlich und das Öffentliche privat sein“ (Knoblauch 2008, S. 6). Das gilt auch für Jana Highholders YouTube-Kanal, wenn sie schreibt: „Dort nehme ich euch mit in mein Leben zwischen Medizinstudium, Bühne, Alltag und zeige, dass Glauben nicht von gestern, sondern für Heute und Morgen ist.“
- Damit verändert sich auch die Sicht auf die bereits von Luckmann nuanciert nachgezeichnete Transformationsdynamik religiöser Subjektivierung: In der populären Kultur als Bezugspunkt sieht sich das Subjekt beständig konfrontiert mit (auch) religiösen Symbolen, Themen und Formen sowie der Erwartung, selbst Erfahrungen transzendenter Art zu machen und wiederum aus dem, was es in seinem Innenraum schöpft, etwas in die Kommunikation einzubringen (Knoblauch 2009, S. 270–272). Daher ist die Subjektivierung bei Knoblauch eine doppelte: Das populär-kommunikativ adressierte Subjekt wird öffentlich als Ausgangspunkt von Religiosität auf Inneres angesprochen und wiederum für die thematisch-religiöse Kommunikation als Quelle spiritueller Erfahrungen angefragt. Wohlgemerkt: Diese „meist emotionale Betonung der individuellen Erfahrung von Transzendenz darf nicht mit Individualisierung verwechselt werden, da sie durchaus mit der Bildung von Gemeinschaften verbunden sein kann“ (Knoblauch 2019, S. 339). Diesem Ineinander von Sozialität und Subjektivität entspricht es, dass Jana Highholder als Influencerin „den Menschen dort begegnen [will], wo sie sind“.
- Freilich zeigt die Fortsetzung des zuletzt zitierten Satzes, dass dieser Begegnungsraum für die Vloggerin spezifisch lokalisiert ist: „und in meiner Generation sind das die sozialen Medien“. Als entscheidender Katalysator Populärer Religion fungieren bei Knoblauch die Medienrevolutionen des 20. und 21. Jahrhunderts – wobei die mit der Digitalisierung verbundenen Transformationen besonders einschneidend sind. Wie Foren zu Engelserfahrungen oder Plattformen für digitale Trauerrituale exemplarisch veranschaulichen, ist gerade im Internet eine Entgrenzung auch zwischen der öffentlichen Sphäre und den Bereichen des Privaten unverkennbar, indem ebenso intime wie persönliche Lebensbereiche Gegenstand von öffentlichkeitsgerichteter Kommunikation sind. Damit ändern sich auch die herkömmlichen Strukturen religiöser Autorität und Expertisezuschreibung: An digitaler religiöser Kommunikation Partizipierende sind auf keine kirchenbehördliche oder religionsorganisationale Legitimierung angewiesen. Vielmehr liegt es an Mitteilungsbereitschaft auf einer und Zuschreibung von Glaubwürdigkeit auf anderer Seite, dass religiöse Kommunikation zustande kommt. Auch hier wird eine Grundannahme der klassischen Individualisierungsthese modifiziert: Während Luckmann eher von einer steigenden Distanz und wechselseitigen Entfremdung von religiösen Experten und Laien ausging (Luckmann 2020, S. 105–107), akzentuiert Knoblauch auch hier den Aspekt der Entgrenzung. In diesem Sinne führt sich Jana Highholder auf ihrer Homepage als „Gottes Influencerin“ ein und schließt ihre Selbstvorstellung mit so etwas wie einem persönlichen „Mission Statement“ ab: „Meine Vision ist eine Jugend, die Gott kennt. Eine Jugend mit Gott.“
- Mit der Medialität ist bei Knoblauch ein weiteres Merkmal Populärer Religion verbunden, nämlich ihre Marktförmigkeit. Diese steht bei ihm beispielhaft für eine fortschreitende Entgrenzung von Funktionsbereichen, die es zunehmend schwer macht, zwischen Religiösem und Nichtreligiösem zu unterscheiden. Stattdessen seien wechselseitige Anverwandlungen festzustellen: Dem Kreationismus zugerechnete Themenparks sind nach Disney-Maßstäben gestaltet, in TV-Unterhaltungsshows treten singende Mönche auf. Solche markanten Beispiele illustrieren, wie zum einen sowohl Formen als auch Inhalte aus dem Heiligen Kosmos religiöser Organisationen in andere Bereiche wandern können und wie zum anderen eben solche Organisationen ihrerseits geprägte Formen populärer Kultur in ihren Handlungsbereichen adaptieren. Auch Jana Highholders Homepageauftritt ist nach Marketing-Gesichtspunkten gestaltet, etwa, wenn sie sich als „Autorin von drei Büchern und Gesicht des Youtube Kanals ‚Jana glaubt‘“ vorstellt.
- Erst vor diesem Hintergrund lässt sich die Funktion der Populärkultur in Knoblauchs Konzept präziser bestimmen. In einer hochgradig ausdifferenzierten Gesellschaft wird die Populärkultur „zum wichtigsten Bindeglied zwischen den funktionalen Systemen, und sie ist deswegen auch das Medium, in dem die Religion kursiert, die die Leute interessiert“ (Knoblauch 2009, S. 237). Konkret denkt Knoblauch hier an „[d]ie Themen, die über das Funktionsspezifische hinausreichen […] die gemeinsamen Mythen, Lieder und Idole, die […] über die Milieus und Schichten hinweg bekannt sind und von denen manche sogar […] zum globalen Wissensreservoir zählen“. Für Jana Highholder ist klar, dass dieses Reservoir – wie überhaupt „alles“ – in den „Social Media“ zu finden ist, weshalb die Userinnen und User auch Gott dort entdecken sollen.
- Das führt uns bereits zur letzten Entgrenzung: Die wechselseitige Durchdringung von religiöser Kommunikation und populärer Kultur ist bei Knoblauch keineswegs auf Phänomene außerkirchlicher Spiritualität beschränkt, sondern prägt auch die traditionelle Sozialgestalt kirchlich verfasster Religion. Die von ihm präsentierten Veranschaulichungsbeispiele reichen „vom Engelsglauben über die Eventisierung der religiösen Zeremonie beim Papstbesuch und bei den Weltjugendtagen bis hin zur Aufnahme der Gegenkultur bei den Jesus Freaks“ (Knoblauch 2008, S. 4 f.). An der Rolle der EKD als Projektträgerin von „Jana glaubt“ zeigt sich beispielhaft, dass die christlichen Kirchen bei diesem religiösen Formwandel keine lediglich passive Rolle einnehmen, sondern auch als Akteurinnen Populärer Religion auftreten.

Die bis heute in Kirche und Theologie einflussreiche und zugleich umstrittene Formel der „Kommunikation des Evangeliums“ eignet sich dazu, dieses Engagement verständlich zu machen, gerade auch in seinen Ambivalenzen. Sie wurde in den 1960er-Jahren von Ernst Lange (1981) in Abgrenzung zum Verkündigungsbegriff eingeführt und ist danach in der Praktischen Theologie als Analyse- und Konzeptbegriff unterschiedlich profiliert worden (vgl. als Überblick Domsgen und Schröder 2014).

Auf der einen Seite verweist die Wendung auf die kommunikative Verfasstheit christlicher Religionspraxis. Bereits bei Lange verbindet sich damit eine kirchen- und theologiekritische Pointe: Die Rede von der *Kommunikation *des Evangeliums richtet sich gegen ein hierarchisch-institutionelles Kirchenverständnis. „Kirche konstituiert sich dadurch, dass Menschen ihren Glauben artikulieren.“ (Engemann 2014, S. 27). Damit verlagert sich auch der Fokus von der lehrhaften Ausformulierung des Glaubens hin zu den religiösen Praktiken des Christentums, die, einschließlich ihrer Materialität, ja auch außerhalb der Kirchen als Teil einer Glaubenskultur Gestalt gewinnen. Dabei gilt: Was sich als Evangelium erschließt, „konstituiert sich für die Kommunizierenden erst im Vollzug der Kommunikation“ (Domsgen 2019, S. 257). Sodann ermöglicht der Rückgriff auf den Kommunikationsbegriff den Anschluss an übergreifende Strukturen, Medien und Formen menschlicher Kommunikation. Im vorliegenden Fragezusammenhang empfiehlt sich Knoblauchs Definition von Kommunikation als „das an anderen orientierte körperliche Handeln, das im Vollzug für die Beteiligten etwas bedeutet, indem sie sich reziprok aufeinander und auf diesen Vollzug als einer Objektivierung orientieren“ (Knoblauch 2017, S. 14). Sie ist praktisch-theologisch insofern besonders anschlussfähig, als in ihr die für Glaubenskommunikation konstitutiven Aspekte der Performativität, Reziprozität und Korporalität von Kommunikation hinreichend gewichtet werden. Ferner schließt die Koppelung des Evangeliums mit dem Kommunikationsbegriff die Anerkenntnis ein, „dass für das zentrale, den Charakter der religiösen Praxis des Christentums prägende Geschehen an und mit Menschen *keine Zusatz- und Sonderkonditionen* in Anspruch genommen werden“ (Engemann 2014, S. 25). Vor diesem Hintergrund ist es nur folgerichtig, dass in neueren Inanspruchnahmen dieser Formel die Umstellung auf digitale Medienkommunikation besondere Aufmerksamkeit gefunden hat (vgl. Grethlein 2015a).

Der perspektivischen Weitung über den Kommunikationsbegriff entspricht auf der anderen Seite eine Blickwinkelfokussierung: Mit der Wendung „Kommunikation des *Evangeliums*“ wird bewusst „das christliche Wirklichkeitsverständnis aufgerufen“ (Schröder 2012, S. 10). Die damit verbundenen Ausblendungen sind umso größer und problematischer, je dezidierter die Formel als leitbegriffliche Alternative zum Religionsbegriff profiliert wird (vgl. bes. Grethlein 2015b). Im vorliegenden Kontext hat der Rekurs auf den Evangeliumsbegriff einen entscheidenden Vorteil: Er entspricht dem Selbstverständnis und den Zielsetzungen der zentralen Akteure, ob auf institutioneller oder auf personaler Ebene. Für die EKD wie auch für Jana Highholder selbst ging es bei dem aufwändig produzierten YouTube-Kanal ja nicht im weiten Sinne um religiöse Sinnkonstruktion, Selbstfindung oder Weltdeutung, sondern um christliche Glaubenskommunikation oder – O-Ton Jana Highholder – „um die beste Botschaft der Welt“.

Allerdings deutete sich bereits vielfältig an, dass die Kommunikation des Evangeliums durch die für die Influencer-Angebote auf YouTube charakteristischen Inszenierungslogiken nicht allein im Blick auf die äußere Form der Kommunikationsvollzüge, sondern auch deren inhaltliche Dimension beeinflusst wird. Diese Transformationen können mithilfe des Mediatisierungsansatzes erhellt werden. Dieser analysiert und deutet „den Wandel von Alltag, Kultur und Gesellschaft im Kontext des Wandels der Medien“ (Krotz 2017, S. 14) – und ist in den letzten Jahren verstärkt auch auf religiöse Kommunikation appliziert worden (vgl. pars pro toto Lövheim und Hjarvard 2019; Hoover 2009; Lundby 2017). Das erkenntnisleitende Interesse religionsbezogener Mediatisierungstheorien reicht über die unabweisbare Tatsache hinaus, dass auch religiöse Menschen und Institutionen immer mehr über Medien kommunizieren oder, wie im Zusammenhang des YouTube-Kanals „Jana glaubt“, dezidiert als Medienproduzenten tätig sind. Sie gehen vielmehr davon aus, dass im Zuge der Mediatisierung die Logik der Medien mehr und mehr in die Logik der Religion eingeht, und versuchen, diese Umformungsprozesse zu rekonstruieren. Dabei ist die Mediatisierung des Religiösen beileibe kein Phänomen erst unserer Tage (vgl. Knoblauch 2009, S. 210–215): Bereits Paulus machte sich mit seinen Briefen die Potenziale des römischen Postsystems zunutze. Im Reformationszeitalter war die religiöse Erneuerung eng mit den neuartigen Verbreitungsmöglichkeiten der Printmedien gekoppelt. Im 18. und 19. Jahrhundert wurden dann Zeitschriften und Magazine zur diskursiven Kommunikationsform aufgeklärter Religion. Und mit Billy Grahams „Crusades“, die sehr bewusst als Massenveranstaltungen inszeniert wurden, trat die Kommunikation des Evangeliums dann ins Fernsehzeitalter ein („Electronic Church“).

Eine im vorliegenden Analysekontext hilfreiche Rekonstruktion religiöser Mediatisierungsdynamiken stammt von der Forschungsgruppe um Andreas Hepp, die den Weltjugendtag in Köln unter diesem Gesichtspunkt untersucht hat (Hepp et al. 2009). In ihren Analysen tritt eindrücklich zutage, wie umfassend Religion auf dieser religiösen Großveranstaltung von medialer Kommunikation durchwebt wird. Sie wird auf der Ebene der Teilnehmenden medial vermittelt und auf der Ebene der Veranstaltenden medial inszeniert. Auch wenn der Weltjugendtag schon 15 Jahre zurückliegt, war bereits damals das Handy Schlüsselmedium der teilnehmenden Jugendlichen: Es diente als Kontakt- und Beziehungsmedium, um sich mit anderen Jugendlichen zu verabreden, um Erlebnisse in Bilddateien festzuhalten und mit anderen zu teilen. Aber das Handy fungierte auch als religiöser Zeichenträger: In Form von Displaybildern oder Klingeltönen wurde es als Bekenntnismedium genutzt, mit dem der eigene Glaube bezeugt und die Zugehörigkeit zu einer globalen Glaubensgemeinschaft bekundet wurde. Zugleich führt die Forschungsgruppe vor Augen, wie bewusst und ambitioniert der Weltjugendtag von den Organisierenden als Medienevent inszeniert wurde. Besonders für die Fernsehberichterstattung wurde der Weltjugendtag als Papstereignis personalisiert. Hepp und sein Team fassen zusammen: „Der Papst fungiert damit als ein Symbol der katholischen Kirche, das es gestattet, sowohl das Sakrale als auch das Populäre des heutigen Katholizismus auszudrücken.“ (Hepp et al. 2009, S. 132). Allerdings thematisieren sie auch die mit dieser Strategie verbundenen Risiken: Die verständliche Intention der Kirche, die bestehenden Mediatisierungslogiken für sich zu nutzen, kann de facto auf eine Fremdbestimmung bis hin zur „Unterwerfung“ hinauslaufen (Hepp et al. 2009, S. 134). An dieser Stelle zeigt sich schließlich, dass auch im religionsbezogenen Mediatisierungsdiskurs Wertungen im Spiel sind: Was in der zuletzt zitierten Wertung als „Unterwerfung“ erscheint, wäre in der populärreligiösen Perspektive Knoblauchs als Ausdruck einer gesamtgesellschaftlichen Transformation zu werten, in dem sich die veränderten Wirklichkeitskonstruktionen religiöser Subjekte positiv geltend machen.

Das durchaus spannungsvolle Überschneidungsfeld von religiösen und digitalen Kommunikationslogiken soll im Folgenden am Beispiel des YouTube-Kanals „Jana glaubt“ untersucht werden.^5^ Zunächst werden die populärkulturellen Eigenlogiken von YouTube-Kommunikation skizziert (3.), um vor diesem Hintergrund das spezifische Crossover-Profil des kirchlich initiierten Kanals „Jana glaubt“ schärfer zu erfassen (4). Daran anschließend wird das Zusammenspiel von Populärer Religion, Kommunikation des Evangeliums und Mediatisierung des Religiösen an zwei Materialanalysen exemplarisch untersucht (5.). Eine kurze Ertragszusammenfassung schließt den Untersuchungsgang ab (6.).

## Zur populärkulturellen Logik von YouTube-Kommunikation

Mediennutzungsanalysen wie die JIM-Studie 2020 (mpfs 2020) bestätigen regelmäßig, dass die Videoplattform YouTube seit ihren Anfängen im Frühsommer 2005 kontinuierlich an Popularität gewonnen hat. Der Jugendlichen „liebstes Internetangebot“ (mpfs 2020, S. 36) ist zugleich auch in der deutschen Bevölkerung insgesamt sehr beliebt: Rund die Hälfte von 1000 im Frühjahr 2019 befragten Personen im Alter von 16 bis 69 Jahren nutzen YouTube mindestens mehrmals wöchentlich (statista 2020, S. 16). Bereits diese Zahlen legen es nahe, die Videoplattform mitsamt der darüber zur Verfügung gestellten und rezipierten Videoclips der Populärkultur im oben skizzierten Sinne zuzurechnen, prägen sie doch den (Medien‑)Alltag so vieler Menschen der Gegenwart maßgeblich mit.

Jedoch greift es zu kurz, die Zugehörigkeit zur Populärkultur allein anhand von Nutzungszahlen und somit von der empirisch erhärteten Popularität eines Mediums her zu bestimmen. Vielmehr ist über den faktischen Massenmediencharakter hinaus danach zu fragen, wie Populärkultur näher begriffen und Phänomene wie YouTube ihr entsprechend zugerechnet oder hiervon abgegrenzt werden können. Dabei ist mit John Storey (2017) eine klare Definition von *Populärkultur* als geradezu aussichtsloses Unterfangen anzusehen. Denn abgesehen von einer in Zahlen ausgedrückten Beliebtheit, die Kulturelles plausibel als etwas faktisch *Populär*kulturelles ausweist, erscheinen weitere Klassifizierungen so vielseitig wie uferlos. So kann Populärkultur als übrigbleibender Rest, damit defizitär orientiert als das verstanden werden, was nicht zur Hochkultur zählt: „Populärkultur wird zur Kultur zweiter Wahl für diejenigen, die außer Stande sind, wahre Kultur zu verstehen oder gar entsprechend wertzuschätzen.“ (Storey 2017, S. 28). Oder sie wird pejorativ als „Massenkultur“ abgewertet, somit als „Kultur, die mit hirnloser Passivität konsumiert wird und zu hirnloser Passivität führt.“ Während Storey noch weitere Definitionsversuche expliziert, hält er letzten Endes keine davon für hinreichend.

Zugleich weist er auf zwei prägende Aspekte hin, denen im Umgang mit YouTube-Videos im Allgemeinen und dem Kanal „Jana glaubt“ im Besonderen beträchtliches Erschließungspotenzial eignet: dass nämlich Populärkultur in ihren Phänomenen einerseits stets vor dem Hintergrund eines kontrastierenden Gegenübers als „das Andere“ zu stehen kommt und dass andererseits für die Interpretation einzelner Phänomene der jeweilige Kontext einschließlich geprägter und tradierter Mediennutzungsweisen mitprägend ist.

Wenn hier nun die Videoplattform YouTube als etwas zur Populärkultur Gehöriges im Sinne Storeys verstanden wird, ist entsprechend jenes *Andere*, im Kontrast dazu Stehende, zu skizzieren. Inmitten einer (auch) vom Metaprozess der Mediatisierung geprägten Kultur der Digitalität (vgl. Stalder 2016) erscheint dies bezogen auf YouTube allerdings bereits dadurch erschwert, dass die Videoplattform – technisch betrachtet – eine Infrastruktur bereitstellt, einen leicht zugänglichen Verbreitungsweg, der für allerlei genutzt wird. Dort findet sich sog. klassische Musik ebenso wie Rap und Techno, Jazz und Soul, Rammstein und Max Raabe, Faust-Inszenierungen und Hape Kerkeling, akademische Fachvorträge und zu Schadenfreude animierende Videomitschnitte alltäglicher Malheur-Situationen. Ist von daher YouTube nicht eher als ein Medium bzw. ein Medienpool für sich zu betrachten, in dem sich nahezu alles tummelt – Populärkulturelles ebenso wie das hiervon unterschiedene *Andere*? Dagegen spricht zunächst, dass nicht der Einzelcontent, sondern die gesamte Videoplattform als spezifischer Modus kultureller Präsentations- und Repräsentationspraxis breitenwirksam ist. Populärkultur nur auf einen Teil des Videoangebots zu beziehen, erscheint daher als eine unangemessene Engführung. Die von Knoblauch akzentuierte Integrationsfunktion schlägt hier voll zu Buche. So wie manche Lieder, Fernsehsendungen oder Werbeslogans „einfach jeder kennt“, ist der *Modus* der YouTube-Nutzung durch Milieus, Sozialschichten und Funktionsbereiche hindurch ebenso verbreitet wie selbstverständlich, gleich, ob einzelne Nutzer oder Produzentinnen dort wissenschaftliche Vorträge, Musikclips oder Vlogs wahrnehmen bzw. selbst einstellen. Das *Andere* gegenüber YouTube ist daher ebenfalls modal zu bestimmen: etwa an Raum und Zeit gebundene Theater- und Kinovorstellungen oder (gebührenpflichtige) TV-Senderangebote ohne umfassende Verfügbarkeit auf prinzipiell jedem internetfähigen Gerät, ohne standardisierte Interaktionsmöglichkeiten durch Kommentare – und vor allem ohne die grundsätzliche Möglichkeit für jede und jeden, zu bereits vorhandenen Videos eigene hinzuzufügen („Broadcast Yourself“-Option).

Für die Interpretation auf dieser populärkulturellen Videoplattform begegnender Daten ist indessen der skizzierte Kontext von entscheidender Bedeutung. Denn das gleiche Video – und sei es eine Konzertaufnahme von Rammstein oder den Berliner Philharmonikern – ist bei YouTube anders eingebettet als bei einer Live-Übertragung, in einem käuflichen Speichermedium oder als Teil des Fernsehprogramms: durch die prinzipiell kosten- und grenzenlose Verfügbarkeit (von Werbung und regionalen Beschränkungen abgesehen), durch Kommentierungsoptionen, durch Möglichkeiten der Einbindung in eigene Websites oder Profile, auch in Social Media außerhalb von YouTube. Kurzum: Die Gesamtanlage der Plattform, auf der Nutzerinnen und Nutzer ein Video zu sehen bekommen, ist wesentlicher Bestandteil damit verbundener Kommunikationsprozesse. Dazu gehören die algorithmusbasierten Empfehlungen weiterer Videos am (rechten) Rand ebenso wie die standardmäßige Autoplay-Weiterführung in den nächsten Clip oder die mit der YouTube-Nutzung einhergehenden individuellen und sozialen Gewohnheiten.

## Der YouTube-Kanal „Jana glaubt“ als Crossover-Angebot im Spannungsfeld von Marktförmigkeit und Subjektorientierung

Während YouTube rund 15 Jahre nach seinen Anfängen noch immer als relativ junge Medienausformung gelten kann, haben sich in der Zwischenzeit die Nutzungsmöglichkeiten – etwa im Blick auf die Begrenzung der Dauer von Beiträgen – verändert und bestimmte Videoformate etabliert. In seiner Struktur bildet „Jana glaubt“ vor diesem Hintergrund eine Kombination verschiedener für diese Plattform (inzwischen) typischer Genres. Besonders prägend sind hierbei die Sparten „Wir“, ein thematisch fokussiertes Studioformat, und „Vlog“. Das Genre Vlog (Vlogging/Videoblog) ist als besonders YouTube-spezifisch anzusehen, wird in dieser Variante von „user-created content“ doch der ursprüngliche Slogan der Video-Plattform („Broadcast Yourself“) unmittelbar greifbar. Für YouTube war dieses Format von Anfang an stilbildend (vgl. Burgess und Green 2018, S. 67). Auch gegenwärtig gehören „[t]agebuchartige und bekenntnishafte Aufzeichnungen“ (Haarkötter 2017, S. 133) zu den etablierten Genres der Videoplattform, neben etwa Do-It-Yourself‑, Musik- und News-Videos. Burgess und Green sehen das Phänomen des Vloggings in der Tradition des Varietés („vaudeville“) um die vorletzte Jahrhundertwende:

Vlogging shares this emphasis on liveness, immediacy, and conversation. These characteristics of YouTube’s most prominent genre are essential for understanding the particularity of YouTube, as well as being fundamental to contemporary media entertainment engagement across social media platforms. (Burgess und Green 2018, S. 80 f.)

Bei „Jana glaubt“ hat die EKD mit Mediakraft auf einen YouTube-Profi gesetzt, der als Marktexperte für das Vlog-Genre gilt (vgl. Kneissler 2015). Allerdings sind Vlogs nur eine von mehreren Hauptsäulen von „Jana glaubt“. Ein halbes Jahr nach Einstellung des Kanals finden sich unter den „Top Ten“^6^ drei Vlog-Videos, daneben vier Wir- und drei sonstige Beiträge. Auch auf der Angebotsseite sind Vlogs keineswegs dominant. Von den 185 Jana-Videos sind 79 klar diesem Genre zuzuordnen. Daneben gibt es 63 themenbasierte „Wir“-Studiovideos. Diese sind tendenziell dem Genre Tutorial (vgl. Haarkötter 2017, S. 133) zuzurechnen. Präsentiert werden subjektive Erklärungen, (Handlungs‑)Anleitungen, Ratschläge, Einschätzungen und Positionierungen – teils ausschließlich durch Jana Highholder, teils zusammen mit Gästen gestaltet. Diese Beiträge fokussieren zumeist eine Glaubensfrage und weisen insbesondere dann, wenn die Influencerin alleine vor der Kamera agiert und dabei explizit auf biblische Inhalte Bezug nimmt, auffällige Ähnlichkeiten zur traditionellen Form kirchlicher Evangeliumskommunikation, der Predigt, auf.

Zu den Vlogs und Wir-Videos kommen 43 Videos hinzu, die sich am sinnvollsten unter „Sonstiges“ subsumieren lassen: Sie beinhalten „Q&A“-Clips, in denen Jana Highholder auf Kommentare und Zuschriften aus der „Jana“-Community reagiert, Reportagen über Gefängnisseelsorge oder Schwangerenkonfliktberatung oder sog. Kaffee- bzw. Glühweindates mit in Innenstädten spontan angesprochenen Gesprächspartnern.

Alle drei Video-Kategorien hängen zentral mit der Hauptfigur zusammen: Jana Highholder informiert über Lebens- und Glaubensfragen (Wir), teilt tagebuchartig zahlreiche Erlebnisse ihres Alltags mit (Vlog), interviewt Passantinnen und Passanten oder berichtet als Reporterin. Daher lässt sich von einem Crossover sprechen, das programmatisch populäre YouTube-Genres aufnimmt und kombiniert; das übergreifende Merkmal der starken Personalisierung entspricht der Eigenlogik des Vlogging- bzw. Influencing, insofern Jana Highholder gleichsam als „Marke“ Kristallisationspunkt und Klammer der verschiedenen Videoformate bildet.

Bereits auf der Gattungsebene zeigt sich damit ein spezifisches Mediatisierungsmuster: Auf der einen Seite entspricht die Kommunikation auf dem kirchenfinanzierten YouTube-Kanal erkennbar der Inszenierungslogik von anderen Influencing-Formaten. Das gilt besonders für die Vlog-Beiträge, die sich nicht auf explizite Glaubensthemen beschränken, sondern regelmäßig etwa auch Sport- und Ernährungstipps mit einschließen. Auf der anderen Seite weist der Kanal zugleich ein im Vergleich zu anderen YouTube-Kanälen distinktes Profil auf. Viele der Wir-Videos verfolgen eher traditionelle Motive kirchlicher Evangeliumskommunikation: Manche haben einen stärker religionsdidaktischen Fokus, andere kommen Kurzpredigten nahe, in weiteren geht es um Lebensberatung.

Freilich macht sich die Marktförmigkeit der populär-religiösen User-Orientierung auch bei den Wir-Videos bemerkbar. Es fällt nämlich auf, dass die explizit aufgegriffenen Themen markante Schwerpunkte setzen und gleichzeitig Lücken offenlassen. Während beispielsweise die spezifisch christliche Trinitätslehre in den Video-Titeln^7^ gar nicht begegnet, sind existenzielle Themen, Fragen spiritueller Praxis und Herausforderungen christlicher Lebensführung stark vertreten. Gleich mehrere Beiträge setzen sich mit dem Themenfeld „Gebet“ auseinander, andere mit der Hoffnung auf ein Leben nach dem Tod, mit Vergebung, Neid oder dem persönlichen Umgang mit Zukunftssorgen. Dementsprechend lässt sich die Befassung mit habituellen Gehalten und kognitiven Wissensbeständen des christlichen Glaubens in „Jana glaubt“ als anwendungsorientiert und auf individuelle Adaption fokussiert bezeichnen.

Dabei greift es zu kurz, Marktlogik und Subjektorientierung gegeneinander auszuspielen. Exemplarisch lässt sich dies am Beitrag „DISCO, SAUNA, BIKINIFOTOS – WAS DARF ICH ALS CHRIST TUN?“^8^ (Wir-11) illustrieren. Einerseits dürfte die hohe Nachfrage dieses mit rund 54.000 Klicks drittpopulärsten Kanalvideos durch den unter Marketinggesichtspunkten formulierten Video-Titel mitbedingt sein. Andererseits wird hier ein Fragenkomplex verhandelt, der für junge Christinnen und Christen existenzielle Dringlichkeit besitzt: Gerade hochreligiöse Jugendliche und junge Erwachsene sehen sich mit der Herausforderung konfrontiert, Entfaltungsoptionen der spätmodernen Individualitätskultur mit den Normvorgaben der christlichen Überlieferung auszubalancieren. Eine subjektorientierte Annäherung und eine individuelle Positionierung, wie Jana Highholder sie im Themenvideo präsentiert, dürfte für diese Klientel somit höchst relevant sein. In diese Richtung deuten auch die von mehr als 1500 Userinnen und Usern vergebenen Likes sowie über 400 Kommentare zu diesem Video.

## Exemplarische Detailanalysen

Diese fragmentarischen Beobachtungen zu Kanalvideos auf der Ebene von Themenwahl und Titelformulierungen sollen nun ergänzt werden um eine exemplarische Detailbetrachtung. Diese steht im Zusammenhang mit Jonathan Kühns in Vorbereitung befindlicher Untersuchung zur Kommunikation des Evangeliums in audiovisuellen Online-Formaten. Deren videohermeneutisches Vorgehen lehnt sich analysemethodisch an die Visuelle Wissenssoziologie Jürgen Raabs (2017) an, die – wie in diesem Beitrag – als Basis für weitergehende Mediatisierungsdeutungen dient. Raabs Ansatz begreift sich selbst als empirische Wissenssoziologie und zielt darauf ab, (nach-)produzierte audiovisuelle Daten Dritter methodisch kontrolliert zu verstehen. Gestalterische Komponenten wie unterschiedliche Kadrierungen, Montagen und Spezialeffekte werden dabei ebenso berücksichtigt wie Handlungen vor der Kamera und die Komposition der auditiven und visuellen Elemente. Die Sequenzanalyse bildet das Herzstück des analysierenden Vorgehens und soll in einer Kombination aus gedankenexperimenteller Befremdung und dem Einbringen von Kontextwissen die Fallstruktur des Datums rekonstruieren helfen. Dabei wird ebenso mit Standbildern (Stills) gearbeitet wie mit Partituren, einer tabellenartigen Zwischenstufe zwischen dem vorliegenden Datum und dessen finaler Interpretation in Textform.

In diesem Sinne sollen auch im Folgenden die zu interpretierenden Daten in ihrer komplexen Gesamtkomposition berücksichtigt und ihre Untersuchung nicht auf einzelne Komponenten, wie etwa den gesprochenen Text oder die Ebene des Visuellen allein, verkürzt werden. Konkret in den Blick genommen werden im Weiteren das Standard-Intro in „Jana glaubt“ sowie ausgewählte Teile des Themenvideos „GEBET: FÜHRE ICH SELBSTGESPRÄCHE?“^9^ (Wir-14). Dabei wird deutlich: Weil das Gebet einen Kernvollzug christlicher Glaubenskommunikation darstellt, das in verbalen wie nonverbalen, individuellen wie gemeinschaftlichen Praktiken Gestalt gewinnt, lässt sich die Wechselwirkung zwischen digitaler Medienkommunikation und christlicher Glaubenskommunikation an diesem Beispiel besonders prägnant untersuchen und darstellen. Zugleich ist klar, dass sich Analysebefunde nicht generalisieren lassen. So hätte beispielsweise die Analyse eines der Vlog-Beiträge des Online-Kanals, in denen die Glaubensdimension weniger explizit präsent ist, eine größere Nähe zu anderen Influencing-Formaten ergeben.

### Das Intro

**Figure 1:**
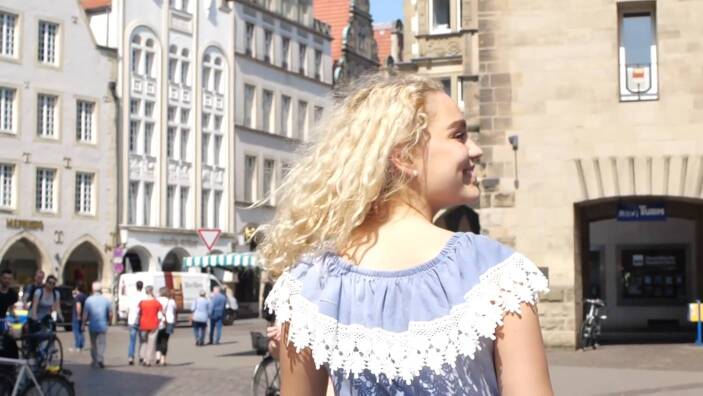

**Figure 2:**
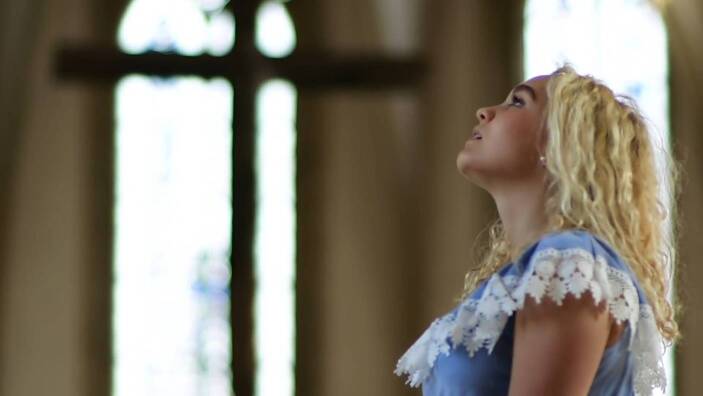

**Figure 3:**
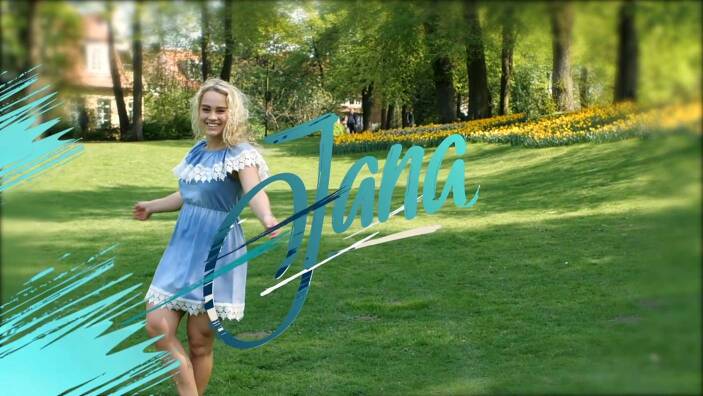

Zu den im YouTube-Kanal „Jana glaubt“ veröffentlichten Videos gehören verschiedene Markensymbole, Identifikationselemente und Plausibilisierungsinstrumente. So enthält das Kennenlernvideo^10^ ein kompaktes „Mission Statement“ des Kanals, in einem Making-of-Clip^11^ wird das Zustandekommen der Studiovideos erläutert, die einzelnen Videobeiträge haben standardmäßig ein festes Intro und enden mit der Schlusseinstellung, die beide grafisch in den Kanalnamen „Jana“ münden (Abb. 3). Im Studio finden eigene „Jana“-Tassen Verwendung, so dass die Hauptfigur wie auch ihre Gesprächspartner das Branding implizit, zuweilen auch explizit befördern. Das Standard-Intro, dem häufig eine kurze Sequenz aus dem nachfolgenden Videokorpus als Teaser vorgeschaltet ist, dauert 13 s. Es besteht aus neun Szenen bzw. bildprägenden Grundeinstellungen der Kamera. Fast im Sekundentakt wechseln die Einstellungen, zeigen Jana Highholder in der Fußgängerzone (Abb. 1), im Gespräch, im Kirchenraum (Abb. 2), am Fluss und im Park (Abb. 3). Den raschen Bildfolgen korrespondieren Inszenierungspraxen der im Zentrum stehenden jungen Frau: Zwar fallen ihre körpereigenen Bewegungen innerhalb der Einzelszenen meist gering aus – nur in der Schlusseinstellung ist sie in einer hüpfenden Drehbewegung im Park zu sehen. Dynamik erwächst aber aus dem Zusammenspiel von häufig wechselnden Schauplätzen und den entsprechend variierenden Körperhaltungen, Blickrichtungen und Kameraperspektiven. Wie der Vorspann einer TV-Serie führt das Intro ein in eine Welt, „Janas“ Welt, in der offenbar Alltag und religiöse Praxis eng miteinander verschränkt sind.

Nonverbal wird eine junge Erwachsene vorgestellt, die mitten im Leben steht, sich souverän durch die Welt manövriert, Lebensfreude und Naturverbundenheit ausstrahlt, zwischenmenschliche Kontakte pflegt und dies alles mit jugendlicher Leichtigkeit tut. Zugleich ist die mosaikartige Sequenzfolge nicht auf das Alltäglich-Diesseitige beschränkt, sondern lässt einen ernsthaften Transzendenzbezug erkennen, wie der andachtsvolle Gestus im Kirchenraum vor dem großen Kruzifix nahelegt (Abb. 2). Lebensfreude und Leichtigkeit gehen einher mit Ernst und Kontemplation. Die inszenierte Mehrbezüglichkeit signalisiert: „Jana glaubt“ vielseitig, in kirchlichen Kontexten und außerhalb, hat einen festen Standpunkt und ist zugleich fortwährend in Bewegung.

Insbesondere die für das Intro gewählten Kameraeinstellungen sorgen für die Einbindung der Zuschauenden ins Geschehen. Teils aus sehr großer Nähe begegnet ihnen Jana Highholder als Gegenüber, auf Augenhöhe; sie werden mitgenommen in ihrem Alltag (Abb. 1). Die Kamera fungiert als Repräsentantin der angesprochenen Internetnutzerinnen und Internetnutzer; sie ist Bindeglied zwischen der Hauptfigur des Kanals und der Community der Adressierten.

Bei den Wir-Videos, die ausschließlich im Studio aufgezeichnet sind, kommt dem Intro eine besonders große Bedeutung zu. Denn es kontextualisiert den sonstigen Clip-Inhalt, rahmt das Format und plausibilisiert, vor welchem lebensweltlichen Hintergrund die junge Frau als „Talking Head“ vor einer Studio-Wohnzimmerwand über ein Thema spricht. Überhaupt wird hier die genretypische Personalisierung noch einmal zugespitzt: Während der Kanal kirchlicherseits unter der Überschrift „Jana glaubt“ lanciert wurde, wird der Kanaltitel im Intro – was auch dem offiziellen Kanalnamen bei YouTube entspricht – ganz ohne Glaubensbezug eingeführt.

### Das Video „GEBET: FÜHRE ICH SELBSTGESPRÄCHE?“

**Figure 4:**
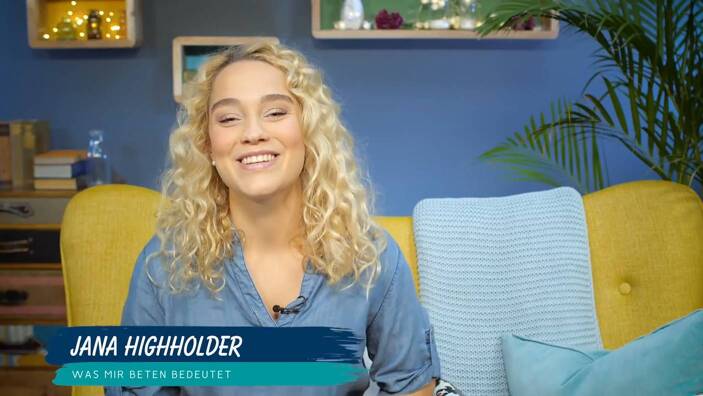

Das Video „GEBET: FÜHRE ICH SELBSTGESPRÄCHE?“ (Wir-14) ist konsequent als Rede konzipiert. Rund acht Minuten lang spricht Jana Highholder im Wohnzimmersetting ohne Gesprächsgegenüber vor der Kamera. Im Vordergrund steht die kommunikative Performance der Hauptakteurin, ergänzt um dezente Klanguntermalung auf der auditiven Ebene. Nur eine Bauchbinde transportiert basale Informationen (Abb. 4); ansonsten wird auf visuelle Illustrationen oder intermediale Elemente wie Videoeinspieler oder Verlinkungen verzichtet. Einzig die häufigen Schnitte und Kadrierungswechsel führen zur visuellen Variation des Gezeigten und brechen die an sich statische Szenerie auf.

**Figure 5:**
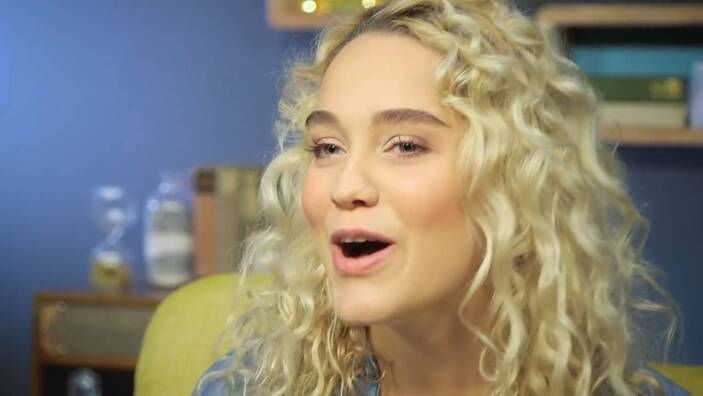

**Figure 6:**
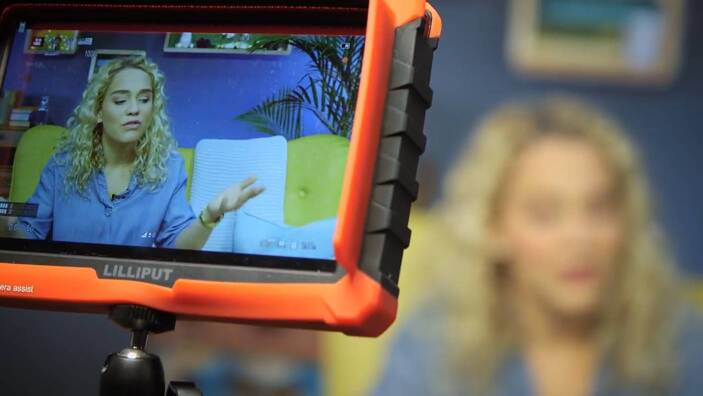

Verglichen mit anderen Studio-Videos – wie etwa Wir-33^12^ mit praktischen Lerntipps – fällt die Variation von Kadrierungen in Wir-14 relativ groß aus und reicht von betonter Distanznahme vom aufgenommenen Geschehen durch ein Bild-im-Bild-Filmen (Abb. 6) bis zu „Big Close Up“-Einstellungen, die Jana Highholders Gesicht oder Hände aus geradezu intimer Nähe zeigen (Abb. 5).

Hierdurch wird visuelle Dynamik in die für YouTube-Verhältnisse lange Verlaufsdauer von rund acht Minuten ohne äußere (Rahmungs‑)Veränderungen gebracht. Durch die gewählten Kameraeinstellungen sowie die optische Nähe des Videobetrachters wird zugleich eine Gesprächssituation inszeniert, die den User oder die Userin als Gegenüber kommunikativ einbindet. Lediglich einmal wird diese Unmittelbarkeit aufgebrochen. Durch einen Bild-im-Bild-Effekt kommt die Medialität der Gesprächskonstellation transparent in den Blick (Abb. 6).

Inhaltsanalytisch lässt sich das Video „GEBET: FÜHRE ICH SELBSTGESPRÄCHE?“ (Wir-14) in drei Teile untergliedern:

1. Vorstellung des Videothemas (TimeCode 00.19–01.08)
2. Erläuterung von Jana Highholders Position und Diskussion von Einwänden (TimeCode 01.09–07.27)
3. Einladung zum Reagieren an die „Jana“-Community (TimeCode 07.28–07.50)

Im Folgenden liegt der Fokus der Analyse auf einigen für die Frage nach dem Wechselverhältnis von *Populärer Religion* und *Kommunikation des Evangeliums* besonders zentralen Aspekten. Das Thema wird in Wir-14 über eine alltagsbezogene Anekdote eingeführt:

Hallöchen, ihr Lieben! Ich freu’ mich, dass ihr wieder dabei seid! Ich möchte euch heute erklären, was Gebet für mich persönlich ist, und auf diese Thematik bin ich gekommen, nachdem ich mit einem sehr guten Freund, der nicht Christ ist und auch, ja, Glauben eher skeptisch gegenübersteht und mit dem ich ganz viel Gesprächsstoff habe, darüber geredet hab’, als ich vor einer Prüfung stand, dass er gesagt hat so: „Hey, ich drück’ dir die Daumen!“ und ich gesagt hab’ so: „Boah, mir wär’s aber viel wichtiger, wenn du für mich beten würdest!“ und diese Diskrepanz zwischen „Hey, ich drück’ dir die Daumen!“ und „Ich bete für dich!“, die war für uns Anlass, mal darüber zu reden, was Gebet überhaupt sein kann (Wir-14, TimeCode 00.27–00.53).

Deutlich stellt auch hier die Personalisierung das Leitprinzip der inhaltlichen Aufbereitung dar. Den Anknüpfungspunkt bietet nicht etwa die Auseinandersetzung mit der biblischen Tradition oder ein Themenwunsch aus der „Jana glaubt“-Community, sondern eine Begebenheit ihres eigenen Studienalltags. Sowohl der Kommunikationsanlass (Prüfung) als auch das kommunikative Setting (Freundeskreis) eröffnen Kontaktflächen zum Alltag der adressierten Zielgruppe aus Jugendlichen und jungen Erwachsenen. Die Ausgangskonstellation ist auch insofern geschickt ausgewählt, als sie unterschiedliche Identifikationen ermöglicht. Hochreligiöse können sich an Jana Highholder orientieren, für die das Gebet einen hohen Stellenwert hat, während dem christlichen Glauben Fernstehende über die Perspektive des „sehr guten“, dem Glauben gegenüber jedoch skeptischen Freundes Zugang finden können.

Die Sequenz steht zudem beispielhaft für ein zentrales Kennzeichen Populärer Religion, nämlich für die Entgrenzung zwischen Privatem und Öffentlichem. Kommunikation des Evangeliums wird öffentlich, indem das Private geteilt wird – in der Erwartung, dass gerade die Darstellung und Mitteilung des Privaten Resonanzen auslöst und den Glauben öffentlich anschlussfähig macht. Besonders deutlich wird diese Entgrenzung im Einstieg. Nach dem Gruß, der rhetorisch eine bereits als etabliert inszenierte Beziehung aktualisiert, wird die Zielperspektive des Videos offengelegt: Jana Highholder will erklären, was das Gebet für sie „persönlich ist“. Nicht die Bekehrung von anderen, sondern die öffentliche Explikation der persönlichen Gottesbeziehung bestimmt hier die digitale Glaubenskommunikation.

Die mit Populärer Religion verbundenen Subjektivierungsdynamiken kommen im weiteren Verlauf des Videos noch deutlicher zum Vorschein. Wieder hat die Argumentation ihren Ausgangspunkt bei subjektiven Glaubenserfahrungen der Vloggerin:

Ich behaupte, dass meine Beziehung zu Gott eine lebendige ist, und ich sage immer wieder, dass ich der Überzeugung bin, dass in jeder Art von zwischenmenschlicher Beziehung Kommunikation ein Schlüssel ist: Freundschaft, Partnerschaft, Teamwork, all diese Dinge leben von Kommunikation, von Austausch miteinander und davon, dass man sich für den anderen interessiert und wissen will, was bei ihm abgeht. Freundschaft funktioniert halt nicht, wenn ich mich nie nach dem anderen erkundige oder nie ihm zuhöre und genauso wenig, wenn ich nie etwas von mir teile. (Wir-14, TimeCode 01.47–02.20)

Der Authentizitätsanspruch des Vorgetragenen wird im Eröffnungssatz durch ein dreifaches Ich verstärkt, wobei der Satzeinstieg der Passage bekenntnishafte Dichte verleiht. Die von Knoblauch namhaft gemachte Emotionalität öffentlich inszenierter religiöser Subjektivität vermittelt sich im Video noch eindrücklicher als im Transkript, über Mimik, Gestik und Klangfärbung. Aufschlussreich ist der anschließende Ebenenwechsel von der Gottesbeziehung zu „jeder Art von zwischenmenschlicher Beziehung“. Kommunikation, so die mit einer Vielzahl privater und beruflicher Identifikationskontexte verknüpfte Pointe, lebt davon, dass man etwas *von sich teilt*. Der mit der für Social-Media-Angebote prägenden Semantik des „Teilens“ hergestellte Anschluss an das populärkulturelle Kommunikationsparadigma wird später im Video explizit, wenn Jana Highholder ausführt: „Ich möchte mit meinem Gott connected bleiben und ich denke, der Weg dazu und dafür ist Gebet, ist Gespräch.“ (Wir-14, TimeCode 03.14–03.23).

Anschließend betont die Influencerin, dass dieses „connected bleiben“ durch gute wie schlechte Zeiten trage – und stellt dann, erstmals, den Bezug auf die biblische Überlieferung her:

Für dieses Wechselspiel der Gefühle sind die Psalme vielleicht das allerbeste Beispiel und irgendwie sind die auch alle ähnlich aufgebaut. David schreibt die meisten von ihnen und David leidet meistens und sagt: „Gott, wo bist du?“ und „Ich seh’ dich nicht!“ und dann irgendwann kommt der Wendepunkt und es wird wieder hell und Gott zeigt sich und sagt: „Hey, ich war da, als du gelitten hast und was du warst ist beharrlich im Gebet auch in den schlechten Zeiten und glaub’ und vertrau’ mir, ich beweise mich immer wieder: Ich bin da und es wird wieder hell!“ (Wir 14, TimeCode 03.50–04.21)

An dieser Stelle bricht erstmals die Spannung zwischen der populär-religiösen Selbstexplikation der Influencerin und der kirchlichen Repräsentationsfunktion ihres Kanals durch. Denn was auf der subjektiven Repräsentationsebene authentisch und stimmig wirkt, wird auf der institutionellen Ebene problematisch. Als theologisch nicht ausgebildete Gläubige freikirchlicher Prägung geht Jana Highholder davon aus, dass die Psalmen direkt von David geschrieben worden sind. Die Gliedkirchen der EKD aber setzen bei den von ihr ordinierten bzw. beauftragten Pfarrerinnen und Pfarrern bzw. Religionslehrkräften ein drei- bis fünfjähriges Theologiestudium voraus. Ein Schwerpunkt liegt dabei auf der historisch-kritischen Bibelinterpretation. Bald schon wurden in der öffentlichen Debatte Stimmen laut, die der EKD vorwarfen, auf ihrem Kanal biblizistische Positionen zu popularisieren (vgl. bspw. Jacobs 2019). Die Spannung lässt sich ebenfalls mit dem Analysekonzept der Populären Religion präziser einordnen: Während der Zugang zur öffentlichen Wortverkündigung in der evangelischen Tradition institutionell autorisiert sein muss und folglich an das Vorhandensein ausbildungsgestützter professioneller Kompetenzen geknüpft ist, erwächst die Autorität Populärer Religion aus der kommunikativen Überzeugungskraft der religiösen Selbstmitteilung. An dieser Stelle zeigt sich, dass die Übersetzung traditioneller Ansprüche der Kommunikation des Evangeliums in die Eigenlogik von YouTube-Formaten mit Aushandlungsprozessen verbunden ist, die kirchlicherseits mit grundsätzlichen Fragen zusammenhängen.

Denn die Pointe dieser kirchlichen Kommunikationsinitiative lag ja auch gerade darin, dass die Hauptperson keine kirchliche Funktionsträgerin ist, sondern eine inszenierungserfahrene Medizinstudentin, die ihren Glauben selbstbewusst lebt und darstellt. Am Ende des Videos wird deutlich, worauf neben Knoblauch auch Felix Stalder (2016, S. 129–164) hingewiesen hat: In der Kultur der Digitalität schließen sich Singularität und Gemeinschaftlichkeit nicht aus, sondern bedingen sich geradezu wechselseitig. Stand bislang die individuelle Gottesbeziehung von Jana Highholder im Vordergrund, wird am Ende Beten als eine gemeinschaftliche Praxis wechselseitiger Vergewisserung eingeführt, mit dem genretypischen Rückverweis auf vergangene Vlogs:

Wenn ich mich zum Beispiel mit Henni und Chris treffe, ihr kennt sie mittlerweile aus meinen Vlogs @(.)@, dann gehen wir selten auseinander, ohne vorher nochmal gemeinsam mit- und füreinander gebetet zu haben für das und über das, was uns auf dem Herzen liegt, was ansteht die neue Woche, Herausforderungen, Dinge, die wir bewältigen müssen oder auch, ja, für Dinge danken, die uns Gutes passiert sind, denn oft ist es so, dass man einem Menschen seine Lasten, Herausforderungen, Aufgaben nicht abnehmen kann, aber es hilft, zu wissen, dass man Menschen hat im Rücken, die einen im Gebet stärken. (Wir-14, TimeCode 06.54–07.27)

Durchaus stimmig wird der Gemeinschaftsbezug am Ende des Videos auf die Community der Rezipientinnen und Rezipienten ausgeweitet. Entsprechend der Grundregel digitaler Kommunikation in Sozialen Medien – nämlich, dass Kommunikation Anschlusskommunikation generieren soll – spielt Jana Highholder vor dem Outro den Ball an die Userinnen und User zurück:

Das war meine Meinung zum Thema Gebet und das, was ich mit Gebet anfange, was Gebet für mich persönlich ist. Wie geht’s euch damit? Was sind eure Erfahrungen mit Gebet? Fällt es euch leichter zu danken, wenn’s gerade gut läuft, oder zu klagen, wenn’s gerade nicht so läuft? Schreibt’s in die Kommentare, berichtet uns davon, und ich freu’ mich, dass ihr nächste Woche oder bei meinem Vlog am Mittwoch wieder dabei seid. (Wir-14, TimeCode 07.27–07.50)

## Ertragszusammenfassung und Diskussion

Die in diesem Beitrag vorgenommene Rahmung, Einordnung und exemplarische Erschließung des von der EKD mitgetragenen YouTube-Kanals „Jana glaubt“ trägt durch folgende Ergebnisse zu der im vorliegenden Sonderheft geführten Diskussion um das Verhältnis von Religion und Populärkultur bei.

Auf einer grundsätzlichen Ebene konnte gezeigt werden, dass sich die von Hubert Knoblauch unter dem Oberbegriff Populärer Religion beschriebenen Phänomene der Entgrenzung bzw. – so die spätere Präzisierung – der „Refiguration“ (Knoblauch 2020) nicht auf Ausdrucksformen, Phänomene und Kontexte kirchlich ungebundener Religiosität beschränken. Sie reichen mittlerweile weit in den Bereich des kirchlichen Christentums, in diesem Fall: des landeskirchlich geprägten Protestantismus in Deutschland, hinein. Vor dem Hintergrund der präsentierten Analysen wäre die Verflüssigung der Grenzen zwischen kirchlich verfasster Religion und Populärer Religion als eine Entgrenzungsdynamik eigener Art zu thematisieren und zu untersuchen. Dabei verläuft diese Dynamik in zwei Richtungen:

Auf der einen Seite wird die Kommunikation des Evangeliums in vielfältiger Weise transformiert, wenn sie nach dem Muster gängiger Influencing-Formate über die digitalen Kommunikationskanäle von YouTube inszeniert wird. Das zeigt sich bereits in der strukturellen Anpassung an das Influencing-Genre und in der Übernahme genretypischer Kommunikationselemente: Wie Internetnutzerinnen und Internetnutzer einem PC-Spiele-Influencer, einer Gesundheitskanal-Figur oder einer Beauty-Expertin „folgen“, so können sie von Jana Highholder etwas (über) Glauben erfahren, durch Kommentare Feedback geben und mit anderen Rezipientinnen und Rezipienten in ein Schreibgespräch eintreten. Die von einer auf Influencer-Marketing spezialisierten Agentur professionell produzierten Videos entsprechen in den Gestaltungselementen und gewählten Gattungen den Standards eines etablierten YouTube-Kanals und stellen durch zahlreiche Interaktionsoptionen Möglichkeiten zur Kommunikation zwischen der Influencerin und ihrer Community bereit. Rückmeldungen, Lob und Kritik, Fragen und Anregungen werden regelmäßig erbeten und zuweilen auch explizit in Videobeiträgen aufgegriffen, besonders deutlich in der Q&A-Sparte.

Über diese eher formale Adaption hinaus konnten tieferliegende Umformungsprozesse rekonstruiert werden, die sich unter Rückgriff auf das Analysekonzept Populärer Religion erfassen ließen: Die Kommunikation des Evangeliums auf dem YouTube-Kanal „Jana glaubt“ ist charakterisiert durch eine starke Personalisierung, bei der Privatsphäre und Öffentlichkeitsbezug, Individualität und Gemeinschaftlichkeit charakteristisch verschränkt werden. Sie wird öffentlich, indem Jana Highholder Einblicke in ihren privaten Alltag gibt, ihren subjektiven Gottesglauben darstellt oder ihre eigene Sichtweise auf Fragen des Glaubens und Lebens mitteilt. Dabei ist digitale Kommunikation nicht nur Medium der Kanalinszenierungen, sondern prägt die Videos auch sprachlich und inhaltlich.

Auf der anderen Seite wirken sich aber auch die Leitperspektive der Evangeliumskommunikation und die kirchliche Einbettung auf die populärreligiösen Darstellungspraktiken aus. So wird dem für das Influencing-Genre typischen Format von Vlogs das tutorialnahe Format der „Wir“-Videos an die Seite gestellt, die einen stärker verkündigenden, pädagogischen oder auch seelsorgerlichen Charakter haben. Sodann treten durch die Entgrenzungsdynamiken zwischen Populärer Religion und kirchlichen Kommunikationsstrategien neuartige Konfliktfelder auf, die im konkreten Fall sowohl die Influencerin als auch die am Kanal beteiligte Institution herausgefordert haben.

Auf zwei zusammenhängende Konfliktfelder soll am Ende dieses Beitrages eigens eingegangen werden. Bereits 2004 sahen Lorne Dawson und Douglas Cowan die etablierten religiösen Institutionen durch den digitalen Wandel in eine gleich doppelte Krise gestürzt: „a crisis of authority and a crisis of authenticity“ (Dawson und Cowan 2004, S. 2). Beide Erschütterungen machen sich auch im untersuchten YouTube-Kanal bemerkbar. Die konsequente Personalisierung und die dadurch von und für Jana Highholder beanspruchte Authentizität (vgl. Radde-Entweiler 2013) stehen mindestens potenziell in Spannung zur redaktionellen Zusammenarbeit mit einer beauftragenden und letztverantwortlichen Instanz wie hier aej/GEP bzw. EKD – und dem Erfordernis, dass am Ende alle Beteiligten einverstanden sein müssen mit den präsentierten Inhalten. Diese Spannung wird im Making-of-Video explizit thematisiert – in mediumstypischer Zuspitzung: „LEGT die KIRCHE mir WÖRTER in den MUND?“ Dass es hier durchaus zu Aushandlungsprozessen kam, gibt die Influencerin selbst zu erkennen: Ihrer Auskunft nach waren in Kanalvideos sowohl solche Inhalte ausgeschlossen, hinter denen sie selbst nicht stehen könnte, wie auch solche, die seitens der Kirche(nvertreter) nicht vertreten werden könnten (Making-of, TimeCode 06.13–06.34).

Die Autoritätsproblematik (vgl. als Überblick Cheong 2013; Hoover 2016) trat wiederum überall dort auf, wo Diskrepanzen zwischen der individuellen Selbstrepräsentation Jana Highholders und den Positionen der EKD aufschienen oder wahrgenommen wurden. An dieser Stelle prallten letztlich doch zwei Welten aufeinander: einerseits die Eigenlogik mediatisierter Populärer Religion, die im Format des Influencings *ganz* auf Personalisierung, Subjekt(ivität) und Authentizität baut – sowie andererseits die in der Öffentlichkeit teilweise vehement geäußerte Erwartung, dass kirchlich beauftragte und finanzierte öffentliche Kommunikation (des Evangeliums) repräsentativ sein oder zumindest doch in umstrittenen Zusammenhängen die (mutmaßliche) Mehrheitsmeinung wiedergeben solle (s. oben Anm. 3). Durch den infolge der kontroversen Diskussion erhöhten Repräsentationsdruck wurde aber gleichzeitig Jana Highholders in dieser Handlungssphäre so fundamentale Anspruch auf Authentizität (vgl. Burgess und Green 2018, S. 42–44) unterminiert.

Dass das Pionierprojekt im Juni 2020 eingestellt wurde, ist nicht mit diesen Spannungen allein zu erklären, hat aber vermutlich auch damit zu tun, dass den in diesem Beitrag untersuchten Entgrenzungsprozessen zwischen Populärer Religion und institutioneller Glaubenskommunikation von beiden Seiten her Widerständigkeit eignet.

## Footnotes

1 Vgl. https://www.wuv.de/tech/die_erfolgreichsten_youtube_videos_des_jahres (05.12.2020).

2 https://www.mediakraft.de/ (05.12.2020).

3 Die vereinzelt artikulierte Kritik an als konservativ eingestuften Einstellungen von Jana Highholder wuchs zu einer öffentlich geführten Kontroverse aus, als zum Weltfrauentag 2019 ein Wir-Video hochgeladen wurde, in dem sie mit Pfarrerin und Kolumnistin Hanna Jacobs die Frage „Müssen sich Frauen unterordnen?“ diskutierte (https://www.youtube.com/watch?v=LUPwelfej54, 28.04.2021). In dem Video erklärt Jana Highholder in einer persönlichen Auslegung von Eph 5,22–24, dass sie sich vorstellen könne, sich einem Mann zu unterstellen, wenn dieser sie liebe oder zu lieben versuche wie Christus die Gemeinde. Besonders der Satz „Ein Mann ist für mich das Oberhaupt der Familie“ führte zu Forderungen, die Evangelische Kirche müsse sich positionell von dem Kanal distanzieren bzw. die Finanzierung ganz einstellen. Zur Diskussion siehe bspw. die im ZEIT-Ressort „Christ & Welt“ unter der Überschrift „Ist sie die Antwort?“ veröffentlichten Beiträge von Hannes Leitlein, Merle Schmalenbach, Erik Flügge, Johann Michael Möller und Hanna Jacobs, https://www.zeit.de/2019/14/jana-highholder-youtube-videos-glaube-jugendliche-evangelische-kirche/komplettansicht (28.04.2021).

4 https://www.janahighholder.de/#uber-mich (17.05.2020).

5 Die YouTube-Videos sind die wichtigste, aber keineswegs einzige digitale Kommunikationsplattform des Projekts „Jana glaubt“. Sie sind vernetzt mit weiteren medialen Kontexten, konkret Instagram und Facebook, auf denen Jana Highholder ebenfalls hochpräsent ist. Auch wenn sich die YouTube-Videos als Herzstück nur künstlich aus diesem Gewebe lösen lassen, ist das Gesamtkonglomerat an digitaler Kommunikation zu komplex, als dass es sich im Rahmen dieses Beitrags abbilden ließe. Daher bleibt die Analyseperspektive im Folgenden auf den Online-Kanal beschränkt.

6 Vgl. https://www.youtube.com/c/Janaglaubt/videos?view=0&sort=p&flow=grid (10.12.2020).

7 https://www.youtube.com/c/Janaglaubt/videos (12.12.2020).

8 https://www.youtube.com/watch?v=LgFd8Da1THU (29.12.2020).

9 https://youtu.be/7mulfcqJy1I (15.12.2020).

10 https://youtu.be/l0M8xbvgCZY (15.12.2020).

11 https://youtu.be/wz_8Dy0ylqU (15.12.2020).

12 Vgl. https://youtu.be/fPhEN3wmwPo 2499.
